# Long Non-Coding RNA in Drug Resistance of Non-Small Cell Lung Cancer: A Mini Review

**DOI:** 10.3389/fphar.2019.01457

**Published:** 2019-12-18

**Authors:** Ruizheng Sun, Ranran Wang, Siyuan Chang, Kexin Li, Rongsi Sun, Mengnan Wang, Zheng Li

**Affiliations:** ^1^ Department of Pathology, Xiangya Hospital, Central South University, Changsha, China; ^2^ NHC Key Laboratory of Carcinogenesis, Cancer Research Institute and School of Basic Medical, Central South University, Changsha, China; ^3^ The Key Laboratory of Carcinogenesis and Cancer Invasion of the Chinese Ministry of Education, Cancer Research Institute, Central South University, Changsha, China; ^4^ Department of Cardiovascular Surgery, The Second Xiangya Hospital, Central South University, Changsha, China

**Keywords:** long non-coding RNAs, drug resistance, non-small cell lung cancer, cisplatin, taxanes, epidermal growth factor receptor tyrosine kinase inhibitors, immune checkpoint inhibitors

## Abstract

Lung cancer is one of main causes of cancer mortality and 83% of lung cancer cases are classified as non-small cell lung cancer (NSCLC). Patients with NSCLC usually have a poor prognosis and one of the leading causes is drug resistance. With the progress of drug therapy, the emergence and development of drug resistance affected the prognosis of patients severely. Accumulating evidence reveals that long non-coding RNAs (lncRNAs), as “dark matters” of the human genome, is of great significance to drug resistance in NSCLC. Herein, we review the role of lncRNAs in drug resistance in NSCLC.

## Introduction

As one of the cancers with high incidence worldwide, lung cancer is the main cause of cancer mortality due to the high fatality related to the disease (Testa et al., 2018). Thirteen percent of lung cancer cases are classified as small-cell lung cancer with 6% relative 5-year survival rate and 83% of cases are non-small cell lung cancer (NSCLC) with 23% 5-year survival rate (Miller et al., 2019). Chemotherapy is the most common treatment for NSCLC and the commonly used drugs include cisplatin (DDP) and the taxanes (Fennell et al., 2016). A study of NSCLC tumor cultures reported that resistance rates to DDP and the taxanes were approximately 63% and 43% (d’Amato et al., 2006). Epidermal growth factor receptor tyrosine kinase inhibitors (EGFR-TKIs) are first-line drugs for advanced NSCLC patients with EGFR-activating mutations (Bruckl et al., 2017; Hirsch et al., 2017). However, EGFR-TKIs only provide around 10–13 months of median progression free survival for NSCLC patients (Tan et al., 2015). Immune checkpoint inhibitors (ICIs) targeting immune-evasive PD-1 axis are also emerging treatments in recent years, but emerging clinical data reports that only 15–25% NSCLC patients responded to ICIs (Zugazagoitia et al., 2017; Pu et al., 2018). Moreover, the drug effect is gradually alleviated with the progress of treatment and is usually impaired by drug resistance (Fennell et al., 2016).

Long non-coding RNAs (lncRNAs) are a set of transcripts with more than 200 nucleotides in length, which are closely related to physiology and pathology process through interacting with DNAs, RNAs, and proteins (Rinn and Chang, 2012; Kopp and Mendell, 2018). Accumulating evidence has proven that the transcriptional dysregulation of lncRNAs plays a critical role in the proliferation, metastasis, and drug resistance of lung cancer (Loewen et al., 2014; Ge et al., 2019; Jiang et al., 2019). Genomic profiling found that altered expression of numerous lncRNAs was closely related to drug resistance in NSCLC. Yang et al. reported that 725 lncRNAs were upregulated and 655 lncRNAs were downregulated in A549/DDP cells compared with in A549 cells (Yang et al., 2013b). Tian et al. established paclitaxel resistant model cells (A549/PTX) and found the upregulation of 119 lncRNAs and downregulation of 1,035 lncRNAs (Tian et al., 2017b). Cheng et al. found 1,731 upregulated and 2,936 downregulated lncRNAs in gefitinib resistant PC9 cell line (Cheng et al., 2015b). Wu et al. identified 703 upregulated and 773 downregulated lncRNAs in gefitinib resistant HCC827-8-1 cells (Wu et al., 2016). Reanalysis of gefitinib, erlotinib, and crizotinib resistance showed the alterations of many lncRNAs in different EGFR-TKIs resistant NSCLC cells and qPCR validated the alterations of four lncRNAs in gefitinib-resistant PC9 cell line (Ma et al., 2017).

LncRNAs mediated drug resistance involves complicated mechanisms. Co-expressed network analysis in drug resistant cells shows that lncRNAs might control resistance through regulating expression of protein-coding genes (Ma et al., 2017). Interactions between lncRNAs and microRNAs (miRNAs) suggests that lncRNAs which harbor miRNAs binding sites can target miRNAs for degradation and mediate drug resistance *via* miRNA sponging effect (Yao et al., 2019). Altered expression of lncRNAs also results in abnormal signaling pathways and regulates effects of anti-cancer drugs (Jiang et al., 2018). Previously, there were several reviews focused on lncRNAs related to DDP and EGFR-TKIs resistance in lung cancer (Chen et al., 2016b; Wang et al., 2018). However, as more drug-resistance relevant lncRNAs become progressively significant because of their aberrant expression, complex biological functions, and potential clinical applications in NSCLC, a thorough and clear review on drug resistance and lncRNAs is warranted for a more comprehensive understanding of different drug resistance mechanisms. Herein, we review the role of lncRNAs in drug resistance to DDP, taxanes, and EGFR-TKIs in NSCLC and summarize lncRNAs and resistance to other drugs targeting abnormally activated signaling pathways and attenuated immune response in NSCLC prospectively.

### LncRNAs and NSCLC DDP Resistance

**Figure 1:**
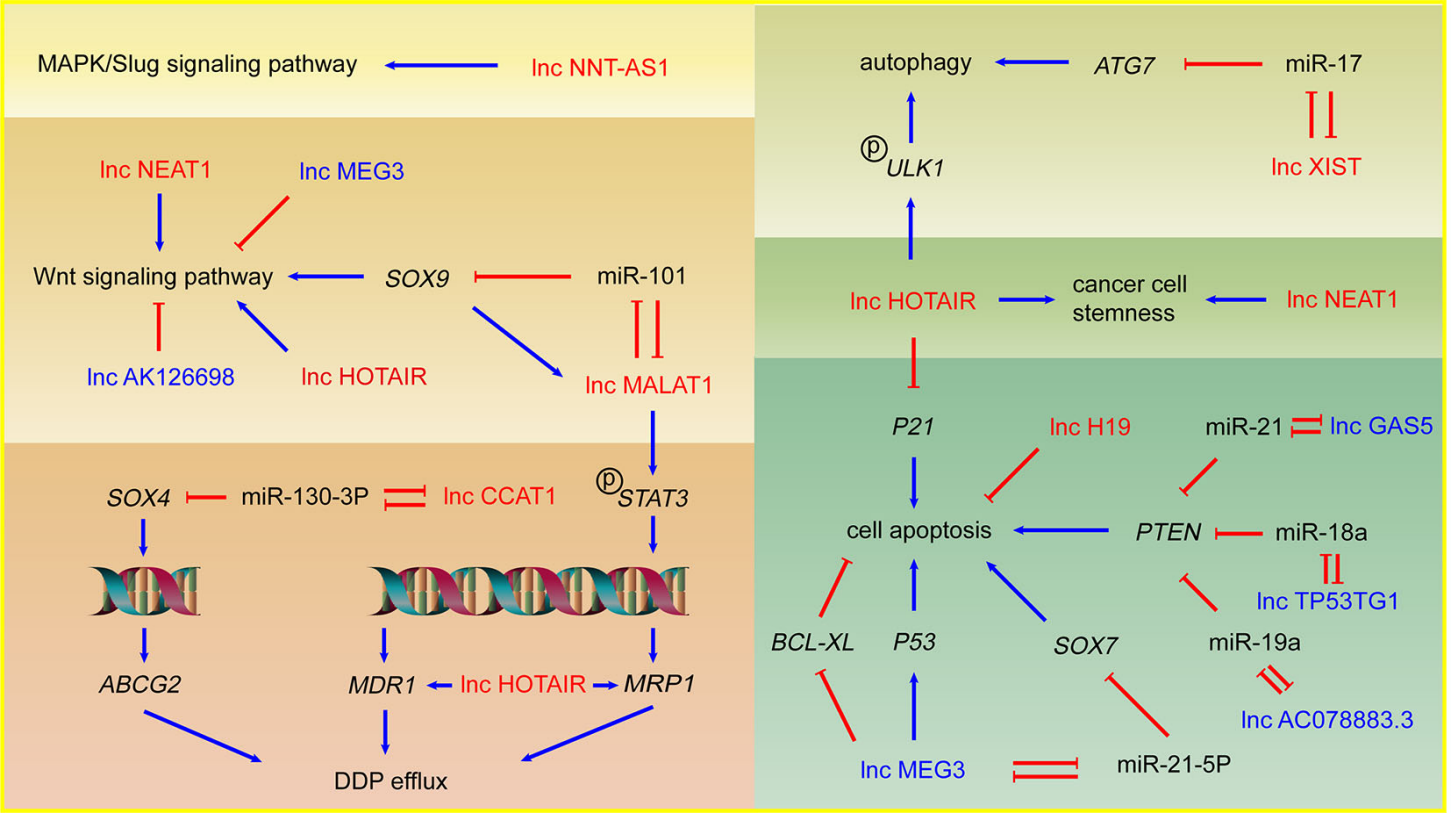
LncRNAs and DDP resistance in NSCLC cells. Arrows in red: promotion; arrows in blue: inhibition; lncRNAs in red: DDP resistance promoting lncRNAs; lncRNAs in blue: DDP sensitivity enhancing lncRNAs.

DDP is the most widely used compound which plays a key role in many cancer treatment programs (Eberhardt et al., 2015). As a kind of alkylating agents, DDP can entry into NSCLC cells to form DNA adducts, induce DNA damage, and result in cell death. The mechanisms of lncRNAs mediated DDP therapeutic effect alteration involve the regulation of several phenotypes such as drug efflux, cell apoptosis, autophagy, cancer cell stemness, etc. through miRNA sponging effect and gene expression regulation. LncRNAs might also regulate DDP resistance or sensitivity in NSCLC *via* controlling Wnt and MAPK/Slug signaling pathway which are closely related to cancer development. Mechanisms involving lncRNAs and DDP resistance are illustrated in **Figure 1**.

### Upregulated LncRNAs in NSCLC DDP Resistance

As one of the biologically well-studied lncRNAs, lncRNA HOTAIR is overexpressed in NSCLC and plays an important role in metastasis (Liu et al., 2013). Recent researches revealed several mechanisms of HOTAIR mediated DDP resistance in NSCLC. Increased expression of energy-dependent translocator helps accelerate DDP efflux in DDP resistant cells. Silencing HOTAIR inhibited the drug transport out of cells through reducing the expression of multidrug resistance 1 (MDR1) and multidrug resistance-associated protein 1 (MRP1) which both belong to the superfamily of ATP Binding Cassette (ABC) transporters and involve drug efflux. HOTAIR may also make a significant contribution to drug resistance by activating Wnt signaling pathway in NSCLC cells (Guo et al., 2018). p21, a cyclin-dependent kinase inhibitor induced by DNA damage, results in cell cycle arrest and inhibition of cell proliferation (Abbas and Dutta, 2009). HOTAIR promotes the DDP resistance in lung adenocarcinoma (LUAD) cells by downregulating p21 protein and overexpressed p21 can rescue the effects of HOTAIR on DDP resistance, which indicates that p21 mediates HOTAIR induced DDP resistance (Liu et al., 2013). Autophagy can be induced by acute DDP treatment and serve as a protective factor to avoid DDP-induced cell death (Galluzzi et al., 2012). Silencing of HOTAIR can suppress phosphorylation of ULK1 to inhibit activation of autophagy, consequently decreasing DDP resistance in NSCLC (Yang et al., 2018). Tumor cell stemness is another important phenotype related to drug resistance, which indicates a difficult cell death induced by DDP and a significant contribution to tumor development and metastasis. HOTAIR could promote DDP resistance in NSCLC cells by upregulating Klf4 which plays an important role in maintaining cell stemness (Liu et al., 2016).

LncRNA MALAT1 is one of the earliest found lncRNAs in NSCLC cells and plays a significant part in tumor development and DDP resistance (Schmidt et al., 2011). Upregulated MALAT1 in DDP resistant NSCLC cell lines serves as sponge of miR-101 and upregulates the target gene SOX9. As transcription factor, SOX9 binds to the MALAT1 promoter and upregulates MALAT1, which forms the MALAT1/miR-101/SOX9 feedback loop. The known downstream of SOX9, Wnt/β-catenin signaling pathway, was involved in MALAT1 mediated DDP resistance (Chen et al., 2017). MALAT1 also activates the transcription factor STAT3, increases the expression of MRP1 and MDR1 *via* STAT3 phosphorylation and promotes NSCLC DDP resistance (Fang et al., 2018).

LncRNA CCAT1 is an oncogene lncRNA significantly upregulated in DDP-resistant NSCLC cells. CCAT1 possibly decreases the sensitivity of NSCLC cells to DDP through CCAT1/miR-130a-3p/SOX4 axis. SOX4 is a target of miR-130a-3p and improves the protein level of ABC Subfamily G Member 2 (ABCG2), which is a transporter protein significant to drug efflux (Hu et al., 2017). LncRNA XIST is upregulated in NSCLC cells and positively associated with the TNM stage (Tantai et al., 2015). XIST is closely associated with NSCLC DDP resistance *via* regulates autophagy through miR-17/ATG7 axis. Knockdown of XIST suppresses NSCLC cells autophagic flux and enhances DDP sensitivity (Sun et al., 2017). LncRNA NEAT1 is a p53-induced lncRNA, playing a key role in cancer progression and NEAT1 is significantly upregulated in A549/DDP cells. Knockdown of NEAT1 inactivates Wnt signaling pathway and downregulates stemness markers, which indicates NEAT1 might play a novel role in stemness and DDP resistance of NSCLC (Mello et al., 2017; Jiang et al., 2018).

LncRNA H19 is significantly overexpressed in DDP resistant NSCLC cells. Knockdown of H19 was related to the upregulation of Fas, Bak, and Bax, which suggests that H19 may promote DDP resistance by regulating cell apoptosis (Wang et al., 2017b). LncRNA NNT-AS1 is upregulated in DDP resistant NSCLC cell lines and tissues. Knockdown of NNT-AS1 results in cell cycle arrest, inhibits cell proliferation, and promotes cell apoptosis *via* downregulation of MAPK/Slug signaling pathway, which suggests MAPK/Slug pathway mediates NNT-AS1 induced DDP resistance in NSCLC (Cai et al., 2018).

### Downregulated LncRNAs in NSCLC DDP Resistance

LncRNA MEG3 is considered as a tumor suppressive gene and downregulated in NSCLC cells with DDP resistance. MEG3 can activate p53 to inhibit the tumor development and enhance the DDP-mediated apoptosis *via* Wnt pathway (Xia et al., 2015). In addition to activating p53, lncRNA MEG3 can improve the apoptosis of the tumor cells by reducing Bcl-xL (Liu et al., 2015). Besides, the transcription factor SOX7 acts as tumor suppressor in NSCLC possibly through activating SPRY1 and SLIT2, and repressing TRIB3 and MTHFD2 (Hayano et al., 2013; Zhang et al., 2018). MEG3 can enhance the sensitivity to DDP of NSCLC *via* MEG3/miR-21-5p/SOX7 axis (Wang et al., 2017a).

LncRNA GAS5 is downregulated in NSCLC cells and improves the sensitivity of NSCLC to DDP lung cancer cells *via* miR-21/PTEN axis (Cao et al., 2017). PTEN is regarded as tumor suppressive regulator in NSCLC and closely associated with DDP resistance (Yang et al., 2013a; Xu et al., 2014). LncRNA TP53TG1 can enhance the sensitivity of NSCLC to DDP *via* miR-18a/PTEN axis (Xiao et al., 2018). Similarly, low expression of lncRNA AC078883.3 contributes to resistance of NSCLC to DDP *via* miR-19a/PTEN axis (Xing et al., 2019b). The study shows that the cells with lower expression of lncRNA AK126698 have stronger resistance to DDP, which main due to Wnt signaling activation (Yang et al., 2013b).

### LncRNAs and NSCLC Taxanes Resistance

Paclitaxel is a natural secondary metabolite isolated from the bark of the gymnosperm yew. Docetaxel, an analog of paclitaxel, inhibits depolymerization of microtubules and mitosis of cells, leads to cell proliferation arrest and achieves the purpose of treating lung adenocarcinoma (Khongkow et al., 2016). However, LUAD with developed taxanes resistance is still the main cause of treatment failure (Du and Morgensztern, 2015). The mechanisms of LncRNAs mediated taxanes resistance mainly focused on modified drug efflux, attenuated cell apoptosis, and enhanced cell proliferation.

LncRNA ANRIL is overexpressed in LUAD tissues and in A549/PTX cells. ANRIL can inhibit apoptosis and induce taxanes resistance *via* Bcl-2 upregulation and cleaved PARP downregulation (Xu et al., 2017). LncRNA CCAT1 serves as the molecular sponge of let-7c and further upregulates Bcl-xL, which enhances proliferation, attenuates apoptosis, and promotes chemical resistance in LUAD cells (Chen et al., 2016a). Linc-ROR serve as sponge of miR-145 leading to overexpression of FSCN1, which induced proliferation of LUAD cells and paclitaxel resistance (Pan et al., 2017).

Ren et al. revealed that lncRNA KCNQ1OT1 expression was upregulated in LUAD tissues and A549/paclitaxel cells. Knockdown KCNQ1OT1 led to MDR1 protein expression decreasing and A549/paclitaxel cells regained the sensitivity to drugs (Ren et al., 2017). However, another study on KCNQ1OT1 revealed that it was upregulated in patients with early-stage NSCLC with good prognosis, acting as an inhibitor of cell proliferation and tumor growth (Sun et al., 2018). These revealed that KCNQ1OT1 might play biphasic functions in NSCLC progression and drug resistance.

### LncRNAs and NSCLC EGFR-TKIs Resistance

**Figure 2:**
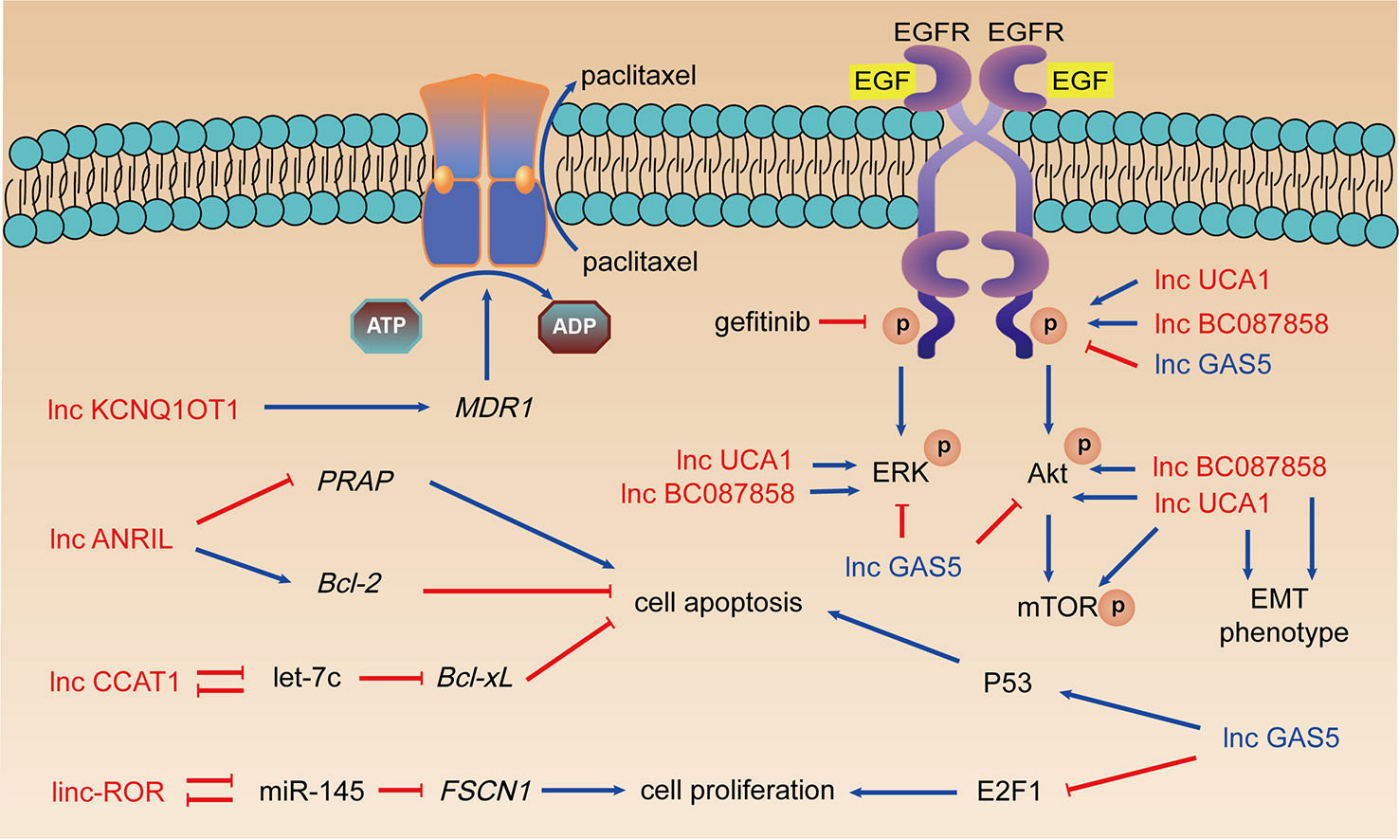
LncRNAs, taxanes resistance, and EGFR-TKIs resistance. Arrows in red: promotion; arrows in blue: inhibition; lncRNAs in red: resistance promoting lncRNAs; lncRNAs in blue: sensitivity enhancing lncRNAs.

EGFR is a transmembrane protein with inherent tyrosine kinase activity, overexpression of which in NSCLC patients is associated with poor prognosis (Ma et al., 2017). NSCLC patients with overexpressed EGFR or mutations in kinase domain of EGFR usually benefit from EGFR-TKIs (Paez et al., 2004). EGFR-TKIs including gefitinib and erlotinib inhibit phosphorylation of EGFR and activation of receptor related kinases. However, long-term treatment usually leads previously sensitive patients to the development of acquired resistance to EGFR-TKIs. LncRNAs regulates EGFR-TKIs resistance or sensitivity through activating EGFR downstream signaling pathway including Akt/mTOR and ERK. Abnormal expression of lncRNAs results in alterations of phenotypes related to EGFR-TKIs resistance including EMT, cell proliferation, apoptosis, etc. (**Figure 2**).

### Upregulated LncRNAs in NSCLC EGFR-TKIs Resistance

Cheng et al. studied the role of lncRNA UCA1 in acquired resistance to EGFR-TKIs in NSCLC, which indicated the overexpression of UCA1 in the PC9/R and H1975 cells (Cheng et al., 2015a). The expression of UCA1 mRNA in NSCLC patients with acquired resistance to gefitinib was obviously increased and connected with the poor prognosis of the patients. Further analysis found that this correlation only existed in NSCLC patients without T790M mutation. Western blot and immunohistochemistry showed that UCA1 was positively connected with p-EGFR, p-ERK, p-AKT, and p-mTOR expression. Silencing UCA1 expression enhances E-cadherin’s expression and weakens expression of vimentin, N-cadherin, and Snail. Therefore, UCA1 may promote gefitinib resistance by activating ERK, AKT/mTOR pathways, and EMT.

LncRNA BC087858 is highly expressed in gefitinib-resistant NSCLC cells. Pan et al. detected the expression of lncRNA BC087858 in different NSCLC cells and EGFR-TKIs resistant tumor tissues, concluding that NSCLC patients with high BC087858 expression had poor prognosis (Pan et al., 2016). This significant correlation also occurred in patients with T790M-mutant negative NSCLC. *In vitro* siRNA-BC087858 transfection can reverse the resistance of T790M-mutant negative NSCLC drug-resistant cells to gefitinib and promote cell apoptosis. BC087858 can promote EMT by up-regulating Snail and ZEB1 expression. It can also activate MEK/ERK and PI3K/AKT signaling pathways by up-regulating p-EGFR, p-ERK, and p-AKT expression, thereby promoting EGFR-TKIs resistance.

### Downregulated LncRNAs in NSCLC EGFR-TKIs Resistance

LncRNA GAS5 is a tumor suppressor and low-expressed in NSCLC. The ectopic expression of GAS5 significantly promoted the expression of p53 and reduced the expression of transcription factor E2F1 which is closely related to cell proliferation (Shi et al., 2015). Dong et al. reported that overexpression of GAS5 could reverse the resistance of A549 cells to gefitinib, by significantly reducing the expression of p-AKT, p-ERK, p-EGFR, and p-IGF1R (Dong et al., 2015). The authors confirmed the results of *in vitro* experiments through *in vivo* experiments, which is a great highlight.

### LncRNAs and NSCLC Resistance to Other Drugs

Rearrangements of anaplastic lymphoma kinase (ALK) in NSCLC were found in 2007 and patients with ALK arrangements benefited a greater improvement of life from ALK-TKIs than from chemotherapy (Soda et al., 2007; Solomon et al., 2014; Katayama et al., 2015). Crizotinib, a multi-targeted ALK/ROS1/MET inhibitor, has been approved by FDA for the treatment of advanced ALK-rearranged NSCLC. However, rapid development of crizotinib resistance usually begins to emerge within 2 years (Doebele et al., 2012). Knockdown of HOTAIR decreased phosphorylation of ULK1, a kinase that is involved with autophagy, which suggested that HOTAIR might promote the drug resistance of NSCLC cells to crizotinib by enhancing autophagy (Yang et al., 2018).

Immunotherapy focuses on improving tumor microenvironment and immune system and ICIs including several monoclonal antibodies targeted PD-1 (nivolumab, pembrolizumab) and PD-L1 (atezolizumab, durvalumab) have been approved by FDA for treatment (Pu et al., 2018). ICIs have better antitumor effect than chemotherapy drugs in NSCLC (Borghaei et al., 2015; Brahmer et al., 2015), but resistance to ICIs are reported with various clinical and molecular features (Gettinger et al., 2018). Xu et al. found 13 lncRNAs closely related to the infiltration of different immune cells (Xu et al., 2018). Among these, lncRNA RP11705C15.3 plays a vital role in the dysregulation of the immune response in most cancer types including NSCLC, which contributed to "avoiding immune destruction" *via* inhibition of T cells (Hanahan and Weinberg, 2011). They also found that dysregulations of lncRNA RP11705C15.3 and SNHG5 are closely related to the prognosis of patients with NSCLC treated with anti-PD-1 immunotherapy. Wei et al. found that the lncRNA MALAT1 had a negative expression correlation with miR-200a-3p and a positive expression correlation with PD-L1 in NSCLC samples. They concluded that MALAT1 could promote the progression of NSCLC *via* miR-200a-3p/PD-L1 axis, which indicated that high expression of MALAT1 in NSCLC might enhance resistance to anti-PD-1 immunotherapy (Wei et al., 2019a). The results indicated that immune-related lncRNAs might have the potential to be biomarkers of immunotherapy response.

### Clinical Prospects of LncRNAs in NSCLC

LncRNAs might have the great potential to be clinical biomarkers which could predict diagnosis and prognosis and indicate the response of drug treatment for NSCLC patients (Navarro et al., 2019; Esfandi et al., 2019; Tan et al., 2017). An increasing number of novel lncRNAs including GAS5, SOX2OT, HOTTIP, OIP5-AS, LINC00473, etc. are potential biomarkers for NSCLC diagnosis and prognosis and should be validated in large NSCLC samples (Chen et al., 2016c; Esfandi et al., 2019; Kamel et al., 2019; Navarro et al., 2019). LncRNA CASC8 rs10505477 could possibly be used to apperceive toxicity and response of chemotherapy in NSCLC patients (Hu et al., 2016). LncRNAs involving tumorigenesis and drug resistance might show a promising future of nucleic acid drug targeting lncRNAs for inhibition of NSCLC progression. LncRNAs targeting therapeutics can be achieved by multiple approaches including RNA interference (RNAi), antisense oligonucleotides (ASOs), morpholinos, CRISPR-Cas9, etc. (Arun et al., 2018). *In vivo* experiments proved that ASOs could significantly target MALAT1 in A549 xenograft model and showed a more effective anti-metastasis response compared with control mice groups (Gutschner et al., 2013). Clinical trials related to NSCLC based on RNAi or ASOs of lncRNA are pursuing although many experiments cannot reach clinical stage for safety (Tian et al., 2017a).

## Conclusions

Accumulating evidence has shown that lncRNAs, as “dark matters” of the human genome, is of great significance to drug resistance in lung cancer. Because of their comprehensive biological functions, especially in regulating gene expression, the relationship between lncRNAs and the drug sensitivity of NSCLC cells has received significant attention. Dysregulation of numerous lncRNAs in drug resistant NSCLC cells has been dug out throughout microarray analyses and lab experiments. In this review, we collect the recently reported experiments *in silico*, *in vitro,* and *in vivo*, aiming to have a better understanding of lncRNAs and drug resistance in NSCLC. Understanding lncRNAs of drug resistance in NSCLC would promote the development of the cognition of diverse factors affecting drug resistance, which would help to break the barrier of drug resistance in NSCLC.

## Author Contributions

RoS, SC, KL, RuS, and MW prepared the manuscript. ZL conceived the idea, reviewed the drafts, and provided important information for the completion of this manuscript. RW reviewed the drafts and provided important information. All authors contributed to the writing and final approval of the manuscript.

## Funding

This study was supported by grants from the Overseas Expertise Introduction Project for Discipline Innovation (111 Project, No. 111-2-12), the National Natural Science Foundation of China (81972773, 81602030), the Hunan Province Natural Sciences Foundation of China (2019JJ40395, 2019JJ40436, 2019JJ40161); and the Undergraduate Training Program for Innovation and Entrepreneurship (201810533372, 201910533180, 20190536).

## Conflict of Interest

The authors declare that the research was conducted in the absence of any commercial or financial relationships that could be construed as a potential conflict of interest.
